# Papular pruritic eruption in HIV‐infected patient: Clinical presentation and noninvasive findings

**DOI:** 10.1111/srt.13191

**Published:** 2022-07-05

**Authors:** Zhe Gao, Xiaofang Zeng, Lifang Hu, Suyang Huang, Yang Li, Xiaohang Du

**Affiliations:** ^1^ Department of Dermatology Hangzhou Third People's Hospital Hangzhou China

**Keywords:** dermoscopy, HIV, hon‐invasive, pruritic papular eruption (PPE), reflectance confocal microscopy

To the Editor,

Pruritic papular eruption (PPE) characterized by obvious pruritus and generalized popular eruptions of unclear etiology is the common cutaneous manifestation in human immunodeficiency virus (HIV) patients. Typically, the diagnosis of PPE is made following clinical manifestations of discrete red papules dominated on trunk and limbs and histological nonfollicular lymphocyte infiltration. The application of noninvasive examination such as reflectance confocal microscopy (RCM) and dermoscopy in diagnosis of PPE has never been reported. Here, we report a case of PPE of a young man with the description of RCM and dermoscopy images.

A 34‐year‐old man was referred to our dermatological department with a 10‐day history of discrete red pruritic papules. Inspection revealed numerous disseminated, peripheral hyperpigmented, erythematous papules (2−5 mm in diameter) on his trunk and extremities (Figures 1A and B). Blood examination in conventional screening revealed positive reaction of HIV, but he had never been diagnosed or treated before. The CD4+ T lymphocyte count (CD4) was 308/mm^3^ (410−1440/mm^3^). The papular lesions were also subjected to potassium hydroxide examination, May‐Grünwald Giemsa stain, and gram stain, and all results came back negative.

**FIGURE 1:**
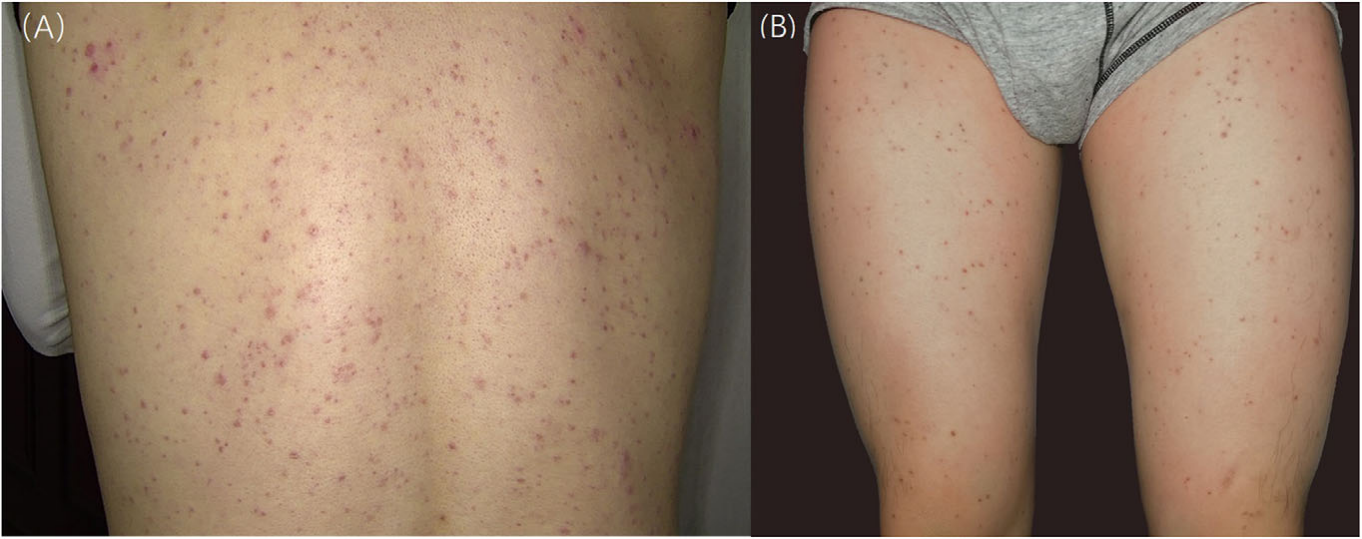
(A, B) Clinical images of PPE revealing erythematous papules on the trunk and extremities

RCM examination of typical rashes showed vasodilation and mild perivascular infiltration of refractive inflammatory cells in the dermal papillae. No significant perifollicular inflammatory cell infiltrate was detected (Figures 2A and B). In the contact polarized mode of the dermoscopy, irregular grayish‐red dotted structures and peripheral hyperpigmentation against a light‐yellow background were observed (Figure 2C). In the polarized noncontact mode, branched starburst‐like vessels were clearly observed and peripheral pigmentation became less pronounced. Notably, branched vessels may be arranged centrally in the hair follicle, but were seen more commonly in nonfollicular areas (Figures 2D and E). At the recent follow‐up examination, we performed a histopathological biopsy from typical rashes. The serial section with hematoxylin‐eosin stain was proved to be consistent with the RCM examination: dermal telangiectasia and lymphocyte‐dominated infiltration were observed. No significant perifollicular inflammatory infiltration was found (Figure 3).

**FIGURE 2:**
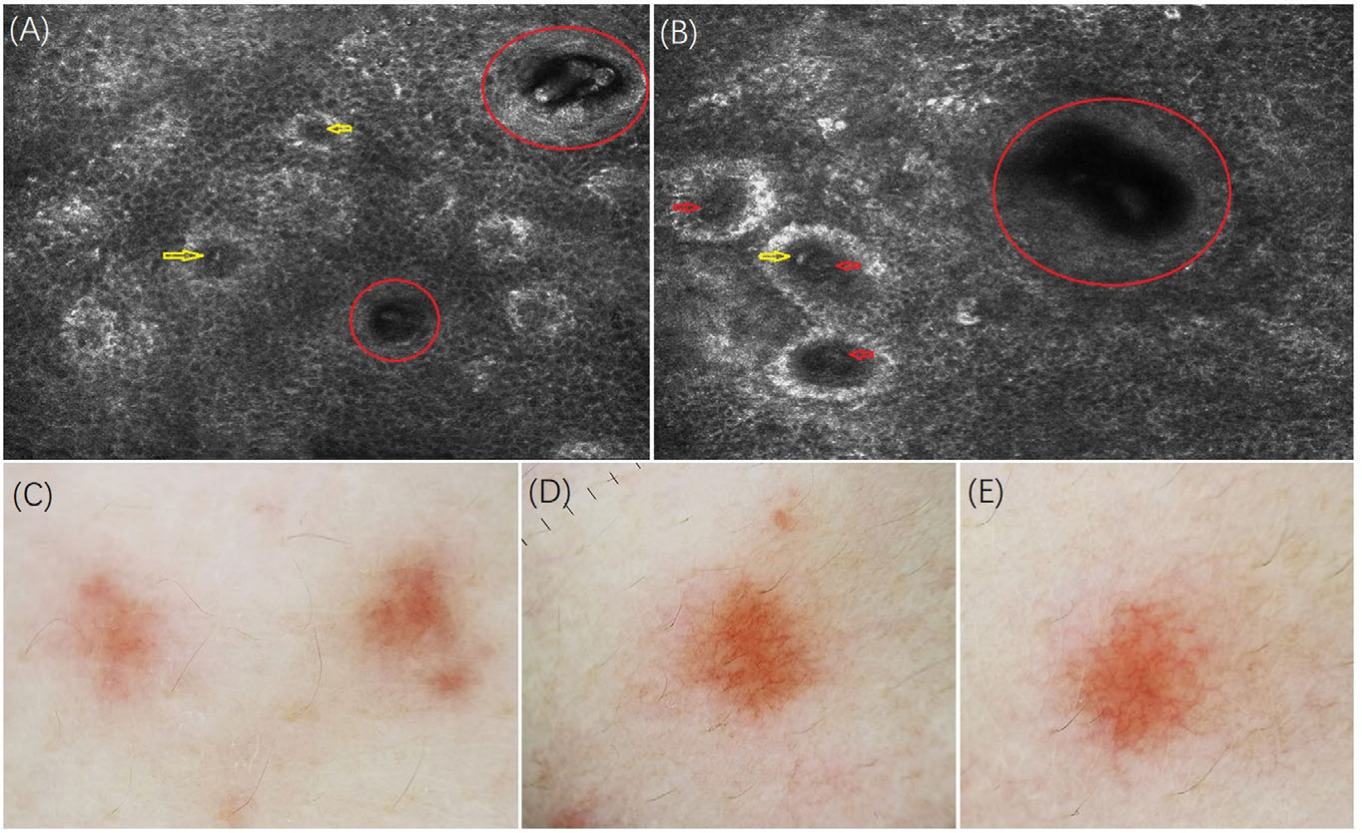
The reflectance confocal microscopy (RCM) images of the prickle cell layer (A) and basal cell layer (B) of back lesions: vasodilation (yellow arrow) and perivascular infiltration of refractive inflammatory cells (red arrow) were observed. No obvious inflammatory cell infiltration was found around the hair follicle (red circle). Dermatoscopy image of back lesions: irregular dark‐red dots and peripheral hyperpigmentation in contact polarized mode (C), starburst‐like vessels and peripheral tan pigmentation in noncontact polarized mode. The vessels can be centered on hair follicles (D) or nonhair follicle areas (E) under dermatoscope

**FIGURE 3:**
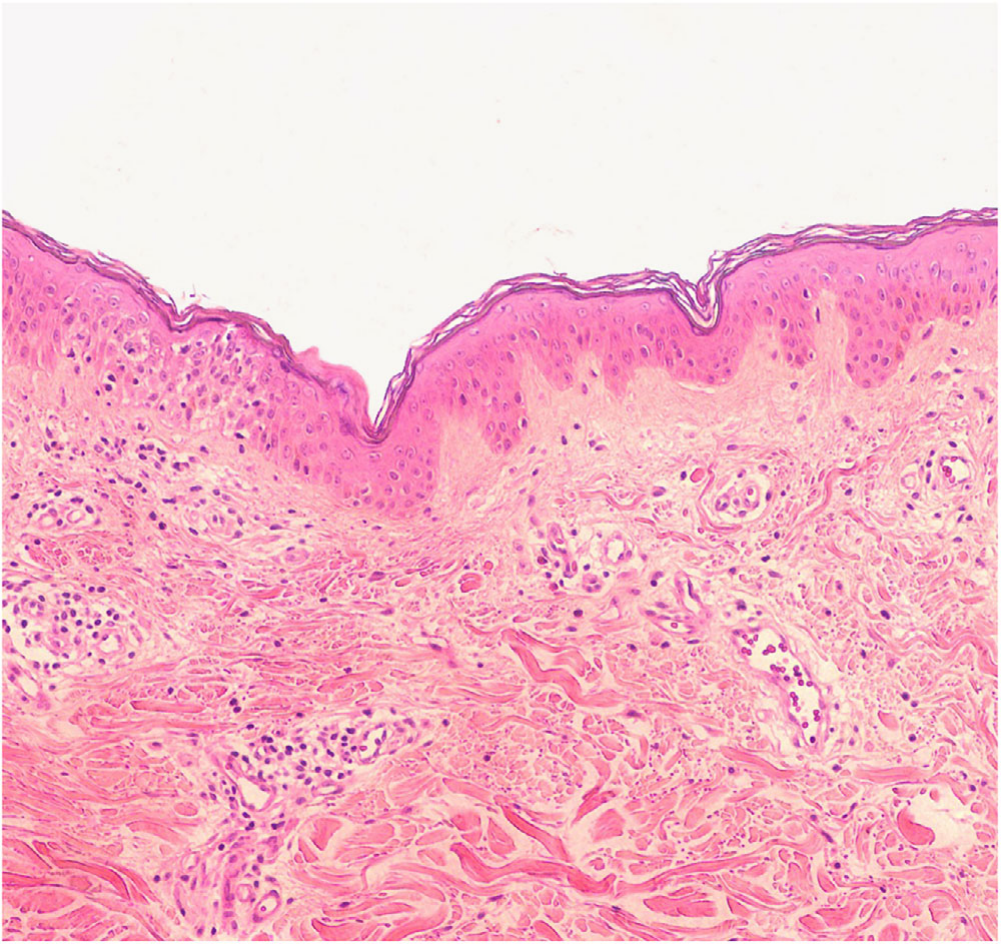
Histopathologic examination revealed focal spongiosis, dermal lymphocyte‐dominated infiltration and vasodilation (Hematoxylin‐eosin stain 100× original magnification)

Skin as the target and effector organ during immune reconstitution is sensitive in HIV infected patients. Numerous HIV‐related dermatological symptoms have been reported in the literature, among which eosinophilic folliculitis (EF) and pruritic papular eruption (PPE) are two confusing entities.^1^, ^2^ HIV‐related EF is an idiopathic eruption characterized by chronic pruritic follicular papules and pustules, preferentially involving seborrheic areas. While PPE is characterized by pruritic hyperpigmented papules that are diffusely distributed on the trunk, extremities, or all over the body.^1^, ^2^, ^3^ Histologically, EF manifests as follicle‐based infiltration of eosinophils with varying admixtures of lymphocytes or histiocytes, whereas PPE often presents spongiosis and perivascular lymphocyte infiltration with occasional eosinophils in the upper dermis.^2^ Possibly due to different inclusion criteria, several large sample studies have shown different results.^1^, ^2^, ^3^, ^4^ Some scholars speculate that HIV‐EF and PPE may be different manifestations in the same disease spectrum during HIV infection.^1^, ^5^ Generally, the key to distinguishing PPE from HIV‐EF is to observe the anatomical distribution and whether the rash is pathological follicle‐centered.

One typical lesion on the back was also sent for biopsy during follow‐up. Pathology was consistent with the RCM examination. Vascular dilatation and lymphocyte‐dominated infiltration were observed in the upper dermis. The lesions were widely distributed on the extremities, and dermoscopy nonfollicular lesions were dominant. Based on the above, this patient was diagnosed with PPE.

Noteworthy, dermoscopy revealed multiple patterns, including branching starburst‐like vessels, postinflammatory hyperpigmentation, folliculocentric lesions, and none folliculocentric lesions. These findings may provide evidence that EF and PPE belong to the same disease spectrum.

It is not difficult to suspect and diagnose PPE when itchy rashes occur in HIV patients. However, in most cases, clinicians do not know whether the first visit patients have HIV infection, which makes PPE easily overlooked. Quick and effective evidence is needed to support the suspicion or confirm the clinician's diagnosis. The description of this case is aimed to provide a potential alternative to skin biopsies and provide helpful clues for understanding of PPE.

## FUNDING/SUPPORT

None.

## Data Availability

The data that support the findings of this study are available on request from the corresponding author. The data are not publicly available due to privacy or ethical restrictions.
